# Influence of single and multiple doses of amifostine on the efficacy and the pharmacokinetics of carboplatin in mice.

**DOI:** 10.1038/bjc.1997.247

**Published:** 1997

**Authors:** A. E. Korst, E. Boven, M. L. van der Sterre, A. M. Fichtinger-Schepman, W. J. van der Vijgh

**Affiliations:** University Hospital Vrije Universiteit, Department of Medical Oncology, Amsterdam, The Netherlands.

## Abstract

We have previously reported that amifostine potentiates the anti-tumour activity of carboplatin in mice. The present study was carried out in well-established human ovarian cancer xenografts OVCAR-3, A2780 and FMa grown subcutaneously in the nude mouse. It was found that a single dose of amifostine resulted in a higher increase in the anti-tumour activity of carboplatin than three doses of amifostine. A single dose of amifostine increased the AUC (area under the curve) values of total platinum in plasma ultrafiltrate (30.1 vs 18.2 microM x h), liver (307.7 vs 236.4 nmol g(-1) x h), kidney (500.8 vs 368.3 nmol g(-1) x h) and OVCAR-3 tumour tissue (184.0 vs 146.8 nmol g(-1) x h). Despite this increase in total platinum, a decrease in platinum (Pt)-DNA adduct levels was observed in liver, kidney and bone marrow, which was significant in liver. In tumour tissue an insignificant increase in Pt-DNA adduct levels, specifically the Pt-GG adduct, was observed after treatment with a single dose of amifostine, which may explain the increase in anti-tumour activity. The increase in the AUC of total platinum was probably caused by a reduction in body temperature, which was most severe after three doses of amifostine. The extreme hypothermia may be the reason that three doses of amifostine resulted in less potentiation of the efficacy of carboplatin.


*British Journal of Cancer* (1997) **75**(10), 1439–1446
© 1997 Cancer Research Campaign

### Influence of single and multiple doses of amifostine on the efficacy and the pharmacokinetics of carboplatin in mice

**AEC Korst[1], E Boven[1], MLT van der Sterre[1], AMJ Fichtinger-Schepman[2] and WJF van der Vijgh[1]**

[1]University Hospital Vrije Universiteit, Department of Medical Oncology, Amsterdam, The Netherlands; [2]TNO Nutrition and Food Research Institute, Rijswijk, The Netherlands

**Summary** We have previously reported that amifostine potentiates the anti-tumour activity of carboplatin in mice. The present study was carried out in well-established human ovarian cancer xenografts OVCAR-3, A2780 and FMa grown subcutaneously in the nude mouse. It was found that a single dose of amifostine resulted in a higher increase in the anti-tumour activity of carboplatin than three doses of amifostine. A single dose of amifostine increased the AUC (area under the curve) values of total platinum in plasma ultrafiltrate (30.1 vs 18.2 μM × h), liver (307.7 vs 236.4 nmol $g^{-1}$ × h), kidney (500.8 vs 368.3 nmol $g^{-1}$ × h) and OVCAR-3 tumour tissue (184.0 vs 146.8 nmol $g^{-1}$ × h). Despite this increase in total platinum, a decrease in platinum (Pt)-DNA adduct levels was observed in liver, kidney and bone marrow, which was significant in liver. In tumour tissue an insignificant increase in Pt-DNA adduct levels, specifically the Pt-GG adduct, was observed after treatment with a single dose of amifostine, which may explain the increase in anti-tumour activity. The increase in the AUC of total platinum was probably caused by a reduction in body temperature, which was most severe after three doses of amifostine. The extreme hypothermia may be the reason that three doses of amifostine resulted in less potentiation of the efficacy of carboplatin.

**Keywords:** amifostine; carboplatin; pharmacokinetics; anti-tumour activity; platinum-DNA adduct; hypothermia

Carboplatin [cis-diammine(1,1-cyclobutanedicarboxylato)platinum(II)] was developed as a second-generation platinum compound with less nephrotoxicity than cisplatin. Its anti-tumour activity is assumed to result from the formation of platinum (Pt)-DNA adducts. The clinical use of carboplatin is limited by myelosuppression at a dose in the steep part of the dose–response curve. Therefore, much effort has been put into reducing the toxic side-effects to allow the administration of higher doses of carboplatin.

Amifostine [Ethyol, WR-2721, S-2-(3-aminopropylamino)-ethylphosphorothioic acid], initially developed as a radioprotector, is approved for use as a protector against chemotherapy-induced toxicities in the USA and Europe (van der Vijgh and Peters, 1994). A selective protection against the side-effects of platinum compounds has been observed in clinical and preclinical studies (Treskes et al, 1992*a*, 1994; Capizzi, 1994; van der Vijgh and Peters, 1994). Amifostine is the prodrug of the aminothiol compound WR-1065 (Figure 1), which inhibits DNA platination (Treskes et al, 1992*b*). The selective protection is based on the preferential formation and uptake of this active metabolite in non-tumour tissues (Yuhas, 1980; Brown et al, 1988; Calabro-Jones et al, 1988; Shaw et al, 1988).

In vitro experiments have shown that the modifying action of amifostine was protection rather than rescue from toxicity (Treskes et al, 1992*b*). Considering these results and the rapid uptake and clearance of WR-1065 by non-tumour tissues after the (i.p. or i.v.) administration of amifostine (Utley et al, 1984; Shaw et al, 1988, 1994), optimal protection would be achieved when amifostine is administered shortly before the platinum drug. Efficient protection against cisplatin-induced nephrotoxicity was observed in mice when amifostine was administered 5 min or 30 min before cisplatin (Treskes et al, 1992*a*). Protection against carboplatin-induced myelotoxicity was more obvious when amifostine was given 5 min instead of 30 min before carboplatin (Treskes et al, 1994). With respect to the long elimination half-life of non-protein-bound carboplatin in comparison with amifostine (van der Vijgh, 1991), greater myeloprotection might be achieved by multiple doses of amifostine when combined with carboplatin. Such an approach is incorporated into clinical trials in which amifostine is given three times, just before and 2 and 4 h after carboplatin (Betticher et al, 1995; Vermorken et al, 1995). To date, clinical data are available for only a small number of patients. The

Amifostine
$H_2N-(CH_2)_3-NH-(CH_2)_2-S-PO_3H_2$

↓ alkaline phosphatase

WR-1065
$H_2N-(CH_2)_3-NH-(CH_2)_2-S-H$

↓ oxidation

Disulphides
$H_2N-(CH_2)_3-NH-(CH_2)_2-S-S-R$

**Figure 1** Structural formulas of amifostine, its active metabolite WR-1065 and the disulphides [with WR-1065 itself or with endogenous thiols (RSH)]

*Received 25 July 1996*
*Revised 28 October 1996*
*Accepted 14 November 1996*

*Correspondence to:* WJF van der Vijgh, University Hospital Vrije Universiteit, Clinical Research Laboratory of Oncology, BR 232, PO Box 7057, 1007 MB Amsterdam, The Netherlands results do suggest that amifostine can reduce the duration of thrombocytopenia and hospitalization (Betticher et al, 1995).

The use of modulating agents can only be successful when these compounds do not interfere with the anti-tumour activity of the cytotoxic agent. Studies in tumour-bearing nude mice have demonstrated that amifostine administered 5 min before the platinum compound did not reduce the anti-tumour efficacy of cisplatin (Treskes et al, 1992*a*) and carboplatin (Treskes et al, 1994). In the case of carboplatin even a potentiation of the anti-tumour activity was noticed when amifostine was given once, whereas amifostine itself did not affect tumour growth (Treskes et al, 1994). However, the influence of multiple doses of amifostine on the anti-tumour activity of carboplatin was not studied.

In the present experiments we investigated the influence of multiple doses of amifostine on the efficacy of carboplatin in tumour-bearing nude mice. We also investigated the influence of one and three doses of amifostine on the pharmacokinetics of carboplatin and the formation of Pt-DNA adducts in order to interpret the efficacy of carboplatin in the presence of amifostine. Furthermore, we determined the influence of a single dose and multiple doses of amifostine on the body temperature in nude mice.

### MATERIALS AND METHODS

### Chemicals

Paraplatin (150 mg of lyophilized carboplatin and 150 mg of mannitol) was obtained from Bristol-Myers Squibb (Woerden, The Netherlands) and reconstituted with 15 ml of glucose 5% before use. Amifostine (500 mg of lyophilized WR-2721 and 500 mg of mannitol) was obtained from US Bioscience (West Conshohocken, PA, USA) and reconstituted with 9.3 ml of sterile water. Before use the compound was further diluted with 0.9% sodium chloride.

### Xenografts and treatment schedules

Female athymic nude mice (Harlan/Cpb, Zeist, The Netherlands), housed and fed as described previously (Boven et al, 1985), were inoculated subcutaneously (s.c.) in both flanks with fragments (2–3 mm in diameter) of the human ovarian cancer xenografts OVCAR-3, A2780 or FMa. OVCAR-3 is a poorly differentiated serous adenocarcinoma, A2780 an undifferentiated carcinoma and FMa a poorly differentiated mucinous adenocarcinoma.

Treatment was started when tumours had reached approximately 300 $mm^3$ in size in the pharmacokinetic study and 50–150 $mm^3$ in the anti-tumour activity studies (designated as day 0). Amifostine injections were given i.p., whereas carboplatin was administered in a tail vein. Drug doses were derived from previous experiments in which carboplatin 60 mg $kg^{-1}$ given on days 0 and 7 was considered the maximum tolerated dose (MTD) allowing 10% weight loss within 2 weeks after the first injection (Treskes et al, 1994). One injection of amifostine (200 mg $kg^{-1}$) given 5 min before carboplatin enhanced the anti-tumour activity in the OVCAR-3 xenograft model. In those experiments the MTD of carboplatin preceded by one injection of 200 mg $kg^{-1}$ amifostine could be increased to 90 mg $kg^{-1}$. In the current study, mice were treated with 60 mg $kg^{-1}$ carboplatin alone or in combination with a single or three doses of 200 mg $kg^{-1}$ amifostine to investigate the influence of amifostine on the anti-tumour activity and pharmacokinetics of carboplatin. Amifostine was administered 5 min before carboplatin as a single dose or 5 min before and 2 and 4 h after carboplatin when administered three times. The 90 mg $kg^{-1}$ dose of carboplatin was also investigated for its anti-tumour activity when administered under the protection of three doses of amifostine.

### Anti-tumour activity

To investigate the influence of three doses of amifostine on the anti-tumour activity of carboplatin, mice bearing bilateral OVCAR-3, A2780 or FMa xenografts (5–6 mice per group) were treated on days 0 and 7 with 60 mg $kg^{-1}$ carboplatin alone or in combination with three times 200 mg $kg^{-1}$ amifostine. A control group (untreated) and a group treated with three doses of amifostine alone were included. In OVCAR-3- and FMa-bearing mice an additional group was treated on days 0 and 7 with 90 mg $kg^{-1}$ carboplatin in combination with three doses of amifostine. In a second experiment the influence of one vs three doses of amifostine on the anti-tumour activity of carboplatin 60 mg $kg^{-1}$ was investigated in mice bearing OVCAR-3 or FMa xenografts, treated on days 0 and 7. In this experiment, additional groups of mice were control (untreated) or treated with carboplatin alone.

In the first experiment mice were weighed twice a week and in the second once (OVCAR-3 and FMa xenografts) or twice (A2780 xenografts) a week. Tumours were measured weekly with a slide caliper by the same observer. Tumour volume was calculated as length × width × height × 0.5 in $mm^3$ and expressed relative to the volume at the start of the treatment. The anti-tumour activity was expressed as the percentage of growth inhibition [1 – (mean of the relative volumes of the treated tumours divided by that of the control tumours) × 100%]. In principle this was calculated on the day of its maximum value, reached within 5 weeks after the last day of treatment. In addition, the number of days for a tumour to reach four times its volume from that at the start of treatment ($T_{D1\rightarrow4}$), was calculated.

### Pharmacokinetics

Mice bearing OVCAR-3 xenografts were treated with 60 mg $kg^{-1}$ carboplatin alone or in combination with 1 × or 3 × 200 mg $kg^{-1}$ amifostine. Of each group, three mice per time point were bled from the axillary vein under ether anaesthesia at 0.5, 1, 1.7, 3, 5, 8, 12 or 24 h after carboplatin administration. Thereafter, liver, kidney and tumours were removed. Bone marrow was collected by flushing both femurs with RPMI medium and pooled from the three mice per time point of each group. The plasma samples were ultrafiltrated by MPS-1 systems provided with YMT filters (Amicon, Capelle a/d IJssel, The Netherlands). Plasma ultrafiltrate and parts of liver, kidney and tumours were stored at –20°C until analysis of total platinum. Bone marrow and parts of liver, kidney and tumours were stored at –80°C until analysis of Pt-DNA adducts.

For platinum analysis the samples were pretreated as follows. Plasma ultrafiltrate samples were diluted 1:1 with 0.15 M sodium chloride/0.4 M hydrochloric acid before measurement of the platinum concentration. Tissue samples of 100 mg were digested in 1.0 ml of concentrated nitric acid in a Teflon bomb at 170°C for 2 h. After cooling, the samples were transferred to glass tubes and evaporated under nitrogen after addition of 20 µl of 1.7 M sodium chloride. The residues were reconstituted in 250 µl of 0.15 M sodium chloride/0.2 M hydrochloric acid. Platinum concentrations were analysed by flameless atomic absorption spectrophotometry using a Spectra AA-300 Zeeman AAS (Varian, Houten, The

© Cancer Research Campaign 1997

**Table 1** Efficacy and toxicity of carboplatin ± amifostine in well-established s.c. human ovarian cancer xenografts in female nude mice ($n \geq 5$ per group) treated weekly × 2 with 3 × 200 mg $kg^{-1}$ amifostine, 60 mg $kg^{-1}$ carboplatin, 60 mg $kg^{-1}$ carboplatin + 3 × 200 mg $kg^{-1}$ amifostine or 90 mg $kg^{-1}$ carboplatin + 3 × 200 mg $kg^{-1}$ amifostine

| Xenograft | Treatment | | Anti-tumour activity | | | Toxicity | | |
|---|---|---|---|---|---|---|---|---|
| | Carboplatin (mg $kg^{-1}$ i.v.) | Amifostine (mg $kg^{-1}$ i.p.) | Growth inhibition (%) (Day) | | $T_{D1\rightarrow4}$ (± s.e.m.)[a] | Maximum weight loss[b] | Weight on day 14[b] | Toxic deaths |
| OVCAR-3 | – | – | NA | NA | 9.9 ± 0.8 | NA | 106.3 ± 3.5 | 0/6 |
| | – | 3 × 200 | 0.0 (28) | 0.0 (35) | 11.0 ± 0.4 | 5.5 ± 2.4 | 105.4 ± 2.3 | 1/6 |
| | 60 | – | 93.2 (28) | 89.0 (35) | 42.8 ± 2.2 | 0.3 ± 2.9 | 104.8 ± 3.8 | 0/5 |
| | 60 | 3 × 200 | 92.2 (28) | 88.2 (35) | 44.4 ± 2.1 | 9.5 ± 2.8 | 98.8 ± 2.0 | 1/6 |
| | 90 | 3 × 200 | 97.7 (28) | 98.2 (35)[c] | 62.7 ± 1.3[c] | 15.9 ± 5.4 | 92.1 ± 4.1 | 1/6 |
| A2780 | – | – | NA | NA | 4.6 ± 0.6 | NA | 118.5 ± 13.4 | 0/6 |
| | – | 3 × 200 | 31.1 (11) | 17.4 (14) | 6.4 ± 1.3 | 4.6 ± 4.8 | 108.0 ± 4.9 | 2/6 |
| | 60 | – | 28.8 (11) | 14.0 (14) | 6.1 ± 0.8 | 0.6 ± 3.2 | 105.6 ± 9.8 | 0/5 |
| | 60 | 3 × 200 | 21.6 (11) | 9.0 (14) | 5.1 ± 1.1 | 11.4 ± 11.7 | 97.6 ± 3.0 | 1/6 |
| FMa | – | – | NA | NA | 12.1 ± 2.3 | NA | 104.9 ± 6.3 | 0/6 |
| | – | 3 × 200 | 20.3 (29) | 21.5 (36) | 13.8 ± 1.3 | 10.7 ± 5.5 | 96.8 ± 3.9 | 0/6 |
| | 60 | – | 90.3 (29) | 89.3 (36) | 46.6 ± 3.2 | 6.6 ± 4.9 | 94.6 ± 7.9 | 0/6 |
| | 60 | 3 × 200 | 93.3 (29) | 92.1 (36) | 46.8 ± 1.8 | 13.5 ± 6.1 | 92.7 ± 8.0 | 0/6 |
| | 90 | 3 × 200 | 97.4 (29) | 98.0 (36)[3] | 60.9 ± 4.3[3] | 20.3 ± 6.4 | 87.1 ± 9.0 | 1/6 |

[a]Time after start of treatment until a relative volume of 4 was reached (days). [b]Calculated as a percentage (± s.d.) of the weight at the start of treatment. [c]$P < 0.02$ compared with 60 mg $kg^{-1}$ carboplatin, $P < 0.01$ compared with 60 mg $kg^{-1}$ carboplatin and 3 × amifostine. NA, not applicable.

**Figure 2** Relative volumes of well-established s.c. OVCAR-3 xenografts grown in female nude mice treated twice (indicated by arrows) with 60 mg $kg^{-1}$ carboplatin i.v. alone (●), in combination with 1× 200 mg $kg^{-1}$ amifostine i.p. (○) or 3 × 200 mg $kg^{-1}$ amifostine i.p. (□) in comparison with control mice (■)

Netherlands). Standards of blank plasma ultrafiltrate and tissue spiked with carboplatin were treated in the same way as the samples.

To determine Pt-DNA adducts, tissue samples were ground to cell suspensions in 2 ml of Tris-EDTA supplemented with 0.5 M ammonium bicarbonate. DNA was isolated as described elsewhere (Blommaert et al, 1995). From the liver samples the cell nuclei were isolated before DNA isolation according to a previously described method (Roggeband et al, 1993). After digestion the isolated DNA samples were chromatographed on a Mono Q anion-exchange column (Pharmacia, Woerden, The Netherlands) and appropriate column fractions were analysed in a competitive enzyme-linked immunosorbent assay (ELISA) (Fichtinger-Schepman et al, 1987). With this procedure four platinum-containing (di)nucleotides could be identified (Fichtinger-Schepman et al, 1987; Blommaert et al, 1995): Pt-GG and Pt-AG (bifunctional adducts of carboplatin with two adjacent guanines or with adenine adjacent to guanine), G-Pt-G [bifunctional adduct of carboplatin with two non-adjacent guanines either in the same strand (intrastrand crosslink) or with two guanines in the opposite DNA strands (interstrand crosslink)] and Pt-G (carboplatin monofunctionally bound to a guanine residue).

Area under the curve (AUC) values of the concentration–time curves of total platinum and of Pt-DNA adducts were calculated from the mean concentrations at each time point, over 0.5–24 h after the carboplatin administration, using the trapezoidal rule.

### Temperature

Non-tumour-bearing nude mice were either injected with 60 mg $kg^{-1}$ carboplatin alone ($n$ = 3) or in combination with 200 mg $kg^{-1}$ amifostine, administered once ($n$ = 3) or three times ($n$ = 3). Temperature changes were monitored for 24 h by measuring body temperature intrarectally by a Laméris Ellab-Instruments thermocouple (Laméris, Utrecht, The Netherlands).

### Statistics

Results of the pharmacokinetic and the anti-tumour activity studies were evaluated with Student's $t$-test.

### RESULTS

### Anti-tumour activity

The anti-tumour activity of carboplatin in the three human ovarian cancer xenografts was expressed as the percentage of growth inhibition and the $T_{D1\rightarrow4}$ (Table 1). Carboplatin at the MTD of 60 mg $kg^{-1}$ was active in OVCAR-3 and FMa xenografts, whereas no significant influence on the growth of A2780 tumours was

© Cancer Research Campaign 1997

**Table 2** Efficacy and toxicity of carboplatin ± amifostine in well-established s.c. xenografts OVCAR-3 and FMa in female nude mice (six per group) treated weekly × 2 with 60 mg $kg^{-1}$ carboplatin alone or in combination with 1 × 200 mg $kg^{-1}$ amifostine or 3 × 200 mg $kg^{-1}$ amifostine

| Xenograft | Treatment | | Anti-tumour activity | | | Toxicity | |
|---|---|---|---|---|---|---|---|
| | Carboplatin (mg $kg^{-1}$ i.v.) | Amifostine (mg $kg^{-1}$ i.p.) | Growth inhibition (%) (Day) | | $T_{D1\to4}$ (± s.e.m.)[a] | Weight on day 14[b] | Toxic deaths |
| OVCAR-3 | – | – | NA | NA | 9.0 ± 0.8 | 109.7 ± 4.7 | 0/6 |
| | 60 | – | 88.5 (20) | 92.4 (27) | 33.5 ± 1.1 | 106.0 ± 2.7 | 0/6 |
| | 60 | 1 × 200 | 92.7 (20)[3] | 96.7 (27) | 43.7 ± 1.4[c] | 104.0 ± 4.0 | 0/6 |
| | 60 | 3 × 200 | 89.9 (20) | 95.2 (27) | 38.8 ± 1.3[d] | 98.3 ± 3.9 | 0/6 |
| FMa | – | – | NA | NA | 11.1 ± 0.8 | 103.2 ± 1.6 | 0/6 |
| | 60 | – | 79.2 (21) | 76.0 (28) | 32.4 ± 2.0 | 95.6 ± 3.4 | 0/6 |
| | 60 | 1 × 200 | 81.5 (21) | 82.0 (28) | 36.8 ± 2.3 | 98.4 ± 2.0 | 0/6 |
| | 60 | 3 × 200 | 81.5 (21) | 78.0 (28) | 35.9 ± 1.9 | 88.2 ± 6.0 | 0/6 |

[a]Time after start of treatment until a relative volume of 4 was reached (days). [b]Calculated as a percentage (± s.d.) of the weight at the start of treatment. [c]$P < 0.001$ compared with 60 mg $kg^{-1}$ carboplatin. $P < 0.02$ compared with 60 mg $kg^{-1}$ carboplatin and 3 × amifostine. [d]$P < 0.01$ compared with 60 mg $kg^{-1}$ carboplatin. NA, not applicable.

**Figure 3** Platinum concentration–time curves and AUC values (from 0.5 to 24 h) in plasma ultrafiltrate (**A**), liver (**B**) kidney (**C**) and OVCAR-3 (**D**) tumour tissue from mice treated with 60 mg $kg^{-1}$ carboplatin alone (●, C) or in combination with 1 × 200 mg $kg^{-1}$ amifostine (○, C + 1A) or 3 × 200 mg $kg^{-1}$ amifostine (■, C + 3A). [1]$P < 0.05$ compared with treatment C; [2]$P < 0.05$ compared with treatment C + 1A

© Cancer Research Campaign 1997

**Figure 4** AUC values of Pt-DNA adducts in liver, kidney, OVCAR-3 tumour tissue and bone marrow from 0.5 to 24 h after treatment with carboplatin alone (C) or in combination with 1 × 200 mg $kg^{-1}$ amifostine (C + 1A) or 3 × 200 mg $kg^{-1}$ amifostine (C + 3A). [1]$P < 0.05$ compared with treatment C; [2]$P < 0.005$ compared with treatment C + 1A

observed. In OVCAR-3 and FMa xenografts the administration of a 1.5-fold increased dose of carboplatin (90 mg $kg^{-1}$), which can only be administered under the protection of amifostine (Treskes et al, 1994), was significantly more effective than 60 mg $kg^{-1}$ carboplatin alone or combined with amifostine ($P < 0.01$). Amifostine, given as a single dose (Treskes et al, 1994) or given three times, did not have any anti-tumour activity. When 60 mg $kg^{-1}$ carboplatin was combined with three doses of amifostine no significant change in growth inhibition was observed when compared with 60 mg $kg^{-1}$ carboplatin alone.

Our results on carboplatin and three doses of amifostine did not correspond with the earlier reported significant potentiation of the anti-tumour activity of carboplatin by one dose of amifostine (Treskes et al, 1994). A second experiment was performed in which we investigated the presence of a dose- or a schedule-dependent interaction between amifostine and carboplatin. Therefore, we examined the influence of one vs three doses of amifostine on the anti-tumour activity of carboplatin in OVCAR-3- and FMa-bearing nude mice (Figure 2, Table 2). When carboplatin was combined with three doses of amifostine a slight increase in the anti-tumour activity was observed in OVCAR-3-bearing mice when compared with carboplatin alone ($T_{D1\rightarrow4}$ 38.8 vs 33.5 days, $P < 0.02$). However, when only one dose of amifostine was administered a more pronounced potentiation of the anti-tumour activity of carboplatin was observed in OVCAR-3-bearing mice when compared with carboplatin alone (growth inhibition 92.7% vs 88.5% and $T_{D1\rightarrow4}$ 43.7 vs 33.5 days, $P < 0.001$), which was comparable to that described before (Treskes et al, 1994). In FMa-bearing mice the same trend was observed, although the difference was not significant (growth inhibition 82.0% vs 76.0% and $T_{D1\rightarrow4}$ 36.8 vs 32.4 days).

As a measure for toxicity, maximum weight loss and the body weight on day 14, both expressed as a percentage of the weight at the start of treatment, as well as the number of toxic deaths have been summarized in Tables 1 and 2. Nadir weight loss after treatment with carboplatin and amifostine was observed 3–4 days after treatment (Treskes et al, 1994). In Table 2 maximum weight loss was not documented, because the weight was measured weekly. In OVCAR-3- and FMa-bearing nude mice the combination of 90 mg $kg^{-1}$ carboplatin and three doses of amifostine was the most toxic (maximum weight loss 15.9% and 20.3% respectively), followed by 60 mg $kg^{-1}$ carboplatin plus three doses of amifostine (9.5% and 13.5% respectively). Amifostine given three times induced more weight loss than when given only once (latter data not shown). Except for the carboplatin 90 mg $kg^{-1}$ plus three doses of amifostine schedule, mice had recovered from weight loss on day 14 of the treatment experiment. In Table 2 it is shown that recovery on day 14 was better in mice receiving carboplatin plus one dose of amifostine than in animals receiving three doses of amifostine. Toxic deaths were observed in mice receiving schedules that contained three doses of amifostine, even without carboplatin. Animals died within 4 days after the injections and this was preceded by excessive weight loss.

### Pharmacokinetics

Concentration–time curves of platinum in plasma ultrafiltrate and tissues from OVCAR-3-bearing nude mice after treatment with carboplatin alone or in combination with one or three doses of amifostine are shown in Figure 3. In plasma ultrafiltrate as well as in normal and tumour tissues, platinum concentrations were higher after treatment with carboplatin and amifostine than with carboplatin alone, resulting in an increase in AUC values as shown in Figure 3. In plasma ultrafiltrate, increases of approximately 1.7-fold and 2-fold were observed when compared with carboplatin alone, of which only the 2-fold increase after three doses of amifostine was statistically significant ($P < 0.05$). In tissues a significant increase of approximately 1.3-fold and 1.5-fold ($P < 0.05$) was observed after treatment with a single and three doses of amifostine, respectively. The difference between the AUC values after the single- and the three-dose schedule was only significant in liver ($P < 0.05$).

The AUC values of Pt-DNA adduct levels are given in Figure 4. Each column shows the AUC value of the total DNA platination, i.e. the sum of the four types of adducts determined in the digested DNA samples. In the normal tissues the total Pt-DNA adduct levels were lower after treatment with a single dose of amifostine than treatment with carboplatin alone. Decreases of approximately 0.8-fold were observed. A significant decrease was only observed in the liver ($P < 0.05$), mainly as a result of decrease in the G-Pt-G adducts. In tumour tissue a slight, insignificant increase of approximately 1.1-fold was seen. When comparing these results with the Pt-DNA adduct levels after treatment with carboplatin and three doses of amifostine, a 1.2- to 1.6-fold increase in total Pt-DNA adducts was observed in normal tissues, which was mainly as a result of higher levels of Pt-G and G-Pt-G adducts. The differences in total Pt-DNA adduct levels between the single- and the three-dose schedules were only statistically significant for the liver samples ($P < 0.005$), whereas significant differences in G-Pt-G levels were found in liver ($P < 0.005$), kidney ($P < 0.01$) and tumour tissue ($P < 0.05$) and in Pt-G levels in liver and kidney ($P < 0.05$).

Because the Pt-DNA adduct levels were not increased proportionally to the increase in total platinum, we calculated the ratio $AUC_{\text{Pt-DNA adducts}}/AUC_{\text{total platinum}}$. In liver tissue this ratio was 0.23 after treatment with carboplatin alone, 0.13 after carboplatin plus one dose of amifostine and 0.18 after carboplatin plus three doses of amifostine. In kidney these ratios were 0.51, 0.31 and 0.40 respectively. In tumour tissue ratios of 0.56, 0.48 and 0.46 were observed for the three treatment schedules.

© Cancer Research Campaign 1997 

**Figure 5** Mean body temperature in non-tumour-bearing nude mice ($n$ = 3 per group) treated with 60 mg kg$^{-1}$ carboplatin alone at $t$ = 0 h (●), in combination with 1 × 200 mg kg$^{-1}$ amifostine (○) or with 3 × 200 mg kg$^{-1}$ amifostine (■)

### Temperature

The mean values of the body temperatures of non-tumour-bearing nude mice after treatment with carboplatin alone, carboplatin in combination with a single dose of amifostine as well as with three doses of amifostine are summarized in Figure 5. Carboplatin alone did not decrease the body temperature of 37°C in the 24 h following injection. After a single dose of amifostine the lowest temperature was 34.5°C, which was observed 1 h after administration. Mice rapidly recovered within 6 h. After three doses of amifostine, however, a mean body temperature of 25°C was observed 7 h after the initiation of treatment, which only started to recover 22 h after the first injection.

### DISCUSSION

Amifostine is an agent that protects against cisplatin-induced nephrotoxicity and carboplatin-induced myelotoxicity without reducing the anti-tumour activity of these platinum compounds in experimental human tumours (Treskes et al, 1992*a*, 1994). The absence of interference with the anti-tumour activity had already been expected from the relatively low reaction rates of carboplatin with amifostine and its main metabolites (Treskes et al, 1991) as well as from the selective uptake of WR-1065 by normal tissues (Calabro-Jones et al, 1988). However, an unexpected potentiation of the anti-tumour activity of carboplatin was observed in nude mice (Treskes et al, 1994), as has also been reported for the combination of amifostine with nitrogen mustard (Valeriote and Tolen, 1982) and that with melphalan (Millar et al, 1982). In 25 patients with non-small-cell lung cancer a high response rate of 64% has been reported upon treatment with cisplatin and vinblastine in combination with amifostine (Schiller et al, 1996), whereas response rates of 25–30% are recorded after standard chemotherapy. In another study in 21 non-small-cell lung cancer patients, treatment with carboplatin and amifostine was at least as active as treatment with carboplatin alone (Betticher et al, 1995).

The influence of amifostine on the anti-tumour activity of carboplatin seemed to be dose- or schedule-dependent in our experiments. When carboplatin was combined with three doses of amifostine no interference with the anti-tumour activity was observed (Table 1). These results did not correspond with the earlier observed significant potentiation of the anti-tumour activity when carboplatin was combined with a single dose of amifostine (Treskes et al, 1994). Our second experiment confirmed that a single dose of amifostine resulted in a significant potentiation of the anti-tumour activity of carboplatin, whereas treatment with three doses of amifostine resulted in a less pronounced increase in the efficacy of carboplatin (Table 2, Figure 2).

From our results on the potentiation of the efficacy of carboplatin by amifostine, we anticipated the presence of a pharmacological drug interaction. In plasma ultrafiltrate, kidney, liver and tumour tissue the platinum concentrations were raised significantly after treatment with amifostine. This pharmacokinetic interaction seemed to be dependent on the treatment schedule. Three doses of amifostine, given just before and 2 and 4 h after the carboplatin administration, resulted in a higher increase in total platinum concentrations than a single dose of amifostine, given just before carboplatin. To investigate the consequence of the increase in total platinum on the supposed target molecule of the anti-tumour drug, DNA in the cell, we determined the Pt-DNA adduct levels in the same tissues and bone marrow.

In the normal tissues a single dose of amifostine resulted in a small decrease in the Pt-DNA adduct levels, mainly due to the level of G-Pt-G adducts, despite the increase in total platinum. Although the observed decrease was significant only in liver, these results might suggest a protection by amifostine as was observed in mice (Treskes et al, 1994). After treatment with three doses of amifostine higher Pt-DNA adduct levels were observed in normal tissues than with a single dose of amifostine, which was most probably due to the higher total platinum levels. These data might indicate that, when amifostine protects against platinum-induced toxicities by reduction of Pt-DNA adduct levels, this protection will be less pronounced after three doses than after a single dose of amifostine. However, this has never been confirmed by toxicity studies, because only the influence of a single dose of amifostine on the carboplatin-induced toxicities has been investigated (Treskes et al, 1994). The small increase in Pt-DNA adduct levels in normal tissues after treatment with three doses of amifostine was not proportional to the rise of the total platinum concentrations, as calculated by the ratio $AUC_{\text{Pt-DNA adducts}}/AUC_{\text{total platinum}}$. The ratios after treatment with carboplatin and amifostine were reduced when compared with the ratio after treatment with carboplatin alone. This relative decrease in Pt-DNA adduct levels was most probably due to the protective properties of amifostine. In vitro studies have already shown that the addition of amifostine or its metabolite WR-1065 to cisplatin resulted in lower Pt-DNA adduct levels (Treskes et al, 1992*b*).

A small but insignificant increase in the amount of Pt-DNA adducts was observed in tumour tissue after treatment with one as well as with three doses of amifostine. Upon calculating the ratio $AUC_{\text{Pt-DNA adducts}}/AUC_{\text{total platinum}}$, a smaller decrease was observed for carboplatin combined with one dose of amifostine than when compared with the ratios in normal tissues. This might indicate that the protective effect of amifostine was mainly restricted to the normal tissues. This is in agreement with the observed selective uptake of WR-1065 in normal tissues when compared with the uptake in tumour tissues (Shaw et al, 1994). As a consequence, no protection of the tumour was to be expected in the efficacy studies, which was indeed the case. Moreover, we even found a potentiation of the anti-tumour activity, especially in the case of one dose of amifostine. Unfortunately, the difference in potentiation between the single and three doses of amifostine was not reflected

© Cancer Research Campaign 1997

by a difference in Pt-DNA adduct levels. This might be due to the very laborious analytical method, which was the only available assay for the detection of the four types of Pt-DNA adducts. The assay turned out to be less precise in detecting small differences. Furthermore, it is questionable whether all four Pt-DNA adducts are responsible for the anti-tumour activity. When the extent of anti-tumour activity was compared with the value of each of the four types of adducts then a positive relation with the major adduct Pt-GG could be established. It is not clear whether amifostine had an influence on the adduct formation or removal when comparing the levels at the individual time points. Owing to the rather high standard deviations differences between the formation and removal of Pt-DNA adducts could not be clearly distinguished.

The reason for the observed amifostine–carboplatin pharmacokinetic interaction and the difference between one and three doses of amifostine added to carboplatin might be the observed amifostine-induced hypothermia. The extreme reduction in body temperature after treatment with three doses of amifostine will result in a peripheral vasoconstriction affecting the renal clearance of carboplatin. This might induce an increase in the AUC values as observed in this study. Although hypothermia leads to a reduction in the cytotoxicity of cisplatin in vitro (Page et al, 1987), no tumour protection was observed in our in vivo experiments. The extreme hypothermic conditions induced by $3 \times 200$ mg $kg^{-1}$ amifostine, however, did result in a less pronounced potentiation of the efficacy of carboplatin. Because hypothermia is related to the dose of amifostine, it also explains the suggested tumour protection by the compound when given as a single dose of 400 mg $kg^{-1}$ (Twentyman, 1983). From other studies in which reduction in body temperature by amifostine has been described in Balb/c (Van der Wilt et al, 1992) and severe combined immunodeficient (SCID) mice (Paine et al, 1996), it appears that the extent of this side-effect varies between different strains of mice. The extreme hypothermia in our nude mice is most probably the reason for the observed toxic deaths (Table 1). The extent of hypothermia in mice is not representative of the human situation because no clear decrease in body temperature has been observed in patients. Therefore, the possible influence of amifostine on the anti-tumour activity of carboplatin still needs to be investigated in patients.

In conclusion, amifostine does not reduce the anti-tumour activity of carboplatin in tumour-bearing nude mice. When given once it even potentiates the anti-tumour activity of carboplatin. Amifostine has an influence on the pharmacokinetics of carboplatin, most probably explained by altered drug distribution caused by hypothermia, resulting in higher platinum concentrations in normal and tumour tissues. The relative reduction in total Pt-DNA adduct levels in these tissues suggests a selective protection of the normal tissues, but not of the tumour tissue. Three doses of amifostine resulted in an extreme reduction of the body temperature as well as toxic deaths and a less pronounced potentiation of the anti-tumour activity. Extrapolating these data to the clinical application of amifostine is difficult because the amifostine-induced hypothermia was not observed in patients. However, our data confirm the suggested selective protection of normal tissues by amifostine, which makes amifostine a promising modulating agent.

### ACKNOWLEDGEMENT

This study was financially supported by the Dutch Cancer Society (IKA 92–104).

### REFERENCES

Betticher DC, Anderson H, Ranson M and Thatcher N (1995) Carboplatin combined with amifostine, a bone marrow protectant, in the treatment of non-small cell lung cancer. *Br J Cancer* **72**: 1551–1555

Blommaert FA, Van Dijk-Knijnenburg HCM, Dijt FJ, Den Engelse L, Baan R, Berends F and Fichtinger-Schepman AMJ (1995) Formation of DNA adducts by the anticancer drug carboplatin: different nucleotide sequence preferences in vitro and in cells. *Biochemistry* **34**: 8474–8480

Boven E, van der Vijgh WJF, Nauta MM, Schlüper HMM and Pinedo HM (1985) Comparative activity and distribution studies of five platinum analogues in nude mice bearing human ovarian carcinoma xenografts. *Cancer Res* **45**: 86–90

Brown DQ, Graham WJ, Mackenzie LJ, Pittock JW and Shaw LM (1988) Can WR-2721 be improved upon? *Pharmacol Ther* **39**: 157–168

Calabro-Jones PM, Aguilera JA, Ward JF, Smoluk GD and Fahey RC (1988) Uptake of WR-2721 derivatives by cells in culture: identification of the transported form of the drug. *Cancer Res* **48**: 3634–3640

Cappizi RL (1994) Protection of normal tissues from the cytotoxic effects of chemotherapy by amifostine (ethyol): clinical experiences. *Semin Oncol* **21**: 8–15

Fichtinger-Schepman AMJ, Van Oosterom AT, Lohman PHM and Berends F (1987) Cis-diamminedichloroplatinum(II)-induced DNA adducts in peripheral leukocytes from seven cancer patients: Quantitative immunochemical detection of the adduct induction and removal after a single dose of cis-diamminedichloroplatinum(II). *Cancer Res* **47**: 3000–3004

Millar JL, McElwain TJ and Clutterbuck RD (1982) The modification of melphalan toxicity in tumor bearing mice by S-2-(3-aminopropylamino)ethylphosphorothioic acid (WR 2721). *Am J Clin Oncol* **5**: 321–328

Page RL, Thrall DE, Dewhirst MW and Meyer RE (1987) Whole body hyperthermia: rationale and potential use for cancer treatment. *J Vet Int Med* **1**: 110–120

Paine GD, Taylor CW, Lopez MHA, Johnson CS and Capizzi RL (1996) Effects of amifostine and paclitaxel on growth of human ovarian carcinoma xenografts in the severe combined immune-deficient mouse: preliminary results. *Semin Oncol* **23** (suppl. 8): 35–39

Roggeband R, Wolterbeek APM, Rutten AAJJL and Baan RA (1993) Comparative $^{32}P$-postlabeling analysis of benzo(a)pyrene-DNA adducts formed in vitro upon activation of benzo(a)pyrene by human, rabbit and rodent liver microsomes. *Carcinogenesis* **14**: 1945–1950

Schiller JH, Storer B, Berlin J, Wittenkeller J, Larson M, Pharo L, Larson M and Berry W (1996) Amifostine, cisplatin and vinblastine in metastatic non-small cell lung cancer: a report of high response rates and prolonged survival. *J Clin Oncol* **14**: 1913–1921

Shaw LM, Glover D, Turrisi A, Brown DQ, Bonner HS, Norfleet AL, Weiler C, Glick JH and Kligerman MM (1988) Pharmacokinetics of WR-2721. *Pharmacol Ther* **39**: 195–201

Shaw LM, Bonner HS and Brown DQ (1994) Metabolic pathways of WR-2721 (ethyol, amifostine) in the Balb/c mouse. *Drug Metab Dispos* **22**: 895–902

Treskes M, Holwerda U, Klein I, Pinedo HM and Van der Vijgh WJF (1991) The chemical reactivity of the modulating agent WR 2721 (ethiofos) and its metabolites with the antitumor agents cisplatin and carboplatin. *Biochem Pharmacol* **42**: 2125–2130

Treskes M, Boven E, Holwerda U, Pinedo HM and Van der Vijgh WJF (1992*a*) Time dependence of the selective modulation of cisplatin-induced nephrotoxicity by WR-2721 in the mouse. *Cancer Res* **52**: 2257–2260

Treskes M, Nijtmans LG, Fichtinger-Schepman AMJ and Van der Vijgh WJF (1992*b*) Effects of the modulating agent WR2721 and its main metabolites on the formation and stability of cisplatin-DNA adducts in vitro in comparison to the effects of thiosulphate and diethylthiocarbamate. *Biochem Pharmacol* **43**: 1013–1019

Treskes M, Boven E, Van de Loosdrecht AA, Wijffels JFAM, Cloos J, Peters GJ, Pinedo HM and Van der Vijgh WJF (1994) Effects of the modulating agent WR2721 on myelotoxicity and antitumour activity in carboplatin-treated mice. *Eur J Cancer* **30A**: 183–187

Twentyman PR (1983) Modification by WR-2721 of the response to chemotherapy of tumours and normal tissues in the mouse. *Br J Cancer* **47**: 57–63

Utley JF, Seaver N, Newton GL and Fahey RC (1984) Pharmacokinetics of WR-1065 in mouse tissue following treatment with WR-2721. *Int J Radiat Oncol Biol Phys* **10**: 1525–1528

Valeriote F and Tolen S (1982) Protection and potentiation of nitrogen mustard cytotoxicity by WR-2721. *Cancer Res* **42**: 4330–4331

Van der Vijgh WJF (1991) Clinical pharmacokinetics of carboplatin. *Clin Pharmacokin* **21**: 242–261

© Cancer Research Campaign 1997

van der Vijgh WJF and Peters GJ (1994) Protection of normal tissues from the cytotoxic effects of chemotherapy and radiation by amifostine (Ethyol): Preclinical aspects. *Semin Oncol* **21**: 2–7

Van Der Wilt CL, Van Laar JAM, Gyergyay F, Smid K and Peters GJ (1992) Biochemical modification of the toxicity and antitumor effect of 5-fluorouracil and cisplatinum by WR-2721 in mice. *Eur J Cancer* **28A**: 2017–2024 .

Vermorken JB, Punt CJA, Eeltink CM, Van Maanen L, Korst AEC, Oster W, Kwakkelstein MO and Van der Vijgh WJF (1995) Phase I trial of carboplatin and amifostine (WR-2721). *Proc AACR* **36**: 240

Yuhas JM (1980) Active versus passive absorption kinetics as the basis for selective protection of normal tissues by S-2-(3-aminopropylamino)ethyl-phosphorothioic acid. *Cancer Res* **40**: 1519–1524

 *© Cancer Research Campaign 1997*


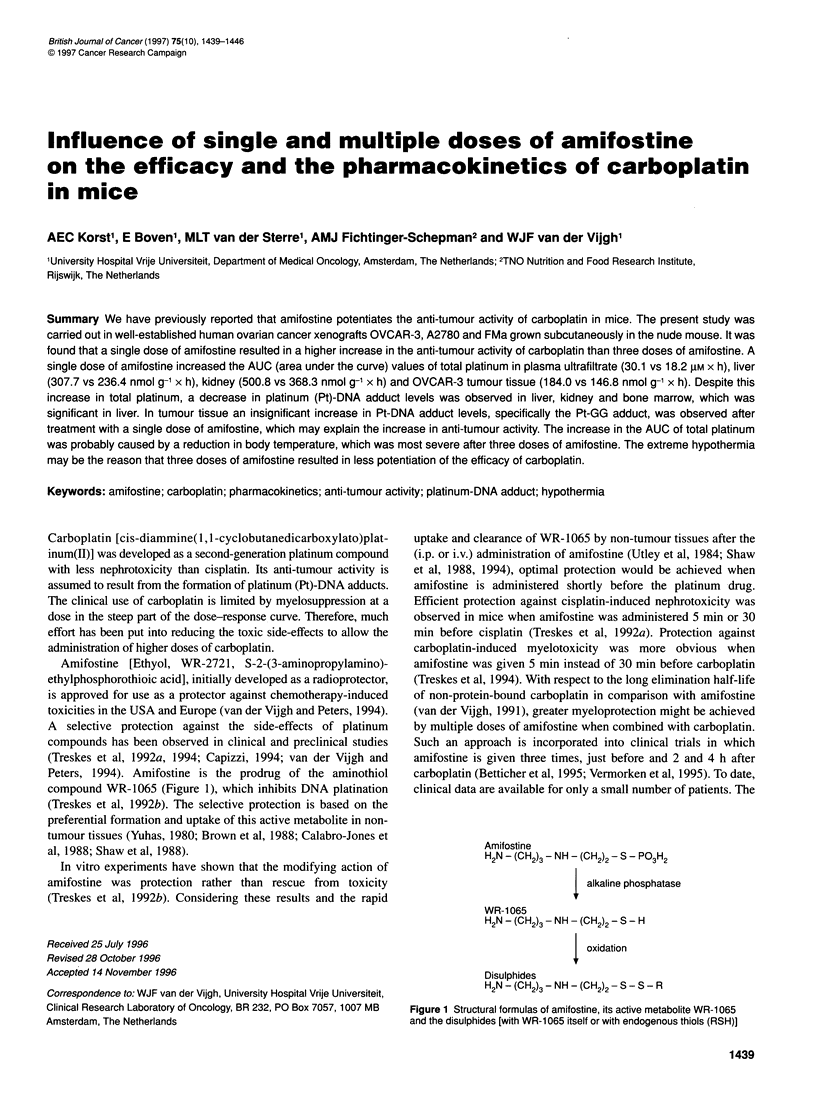

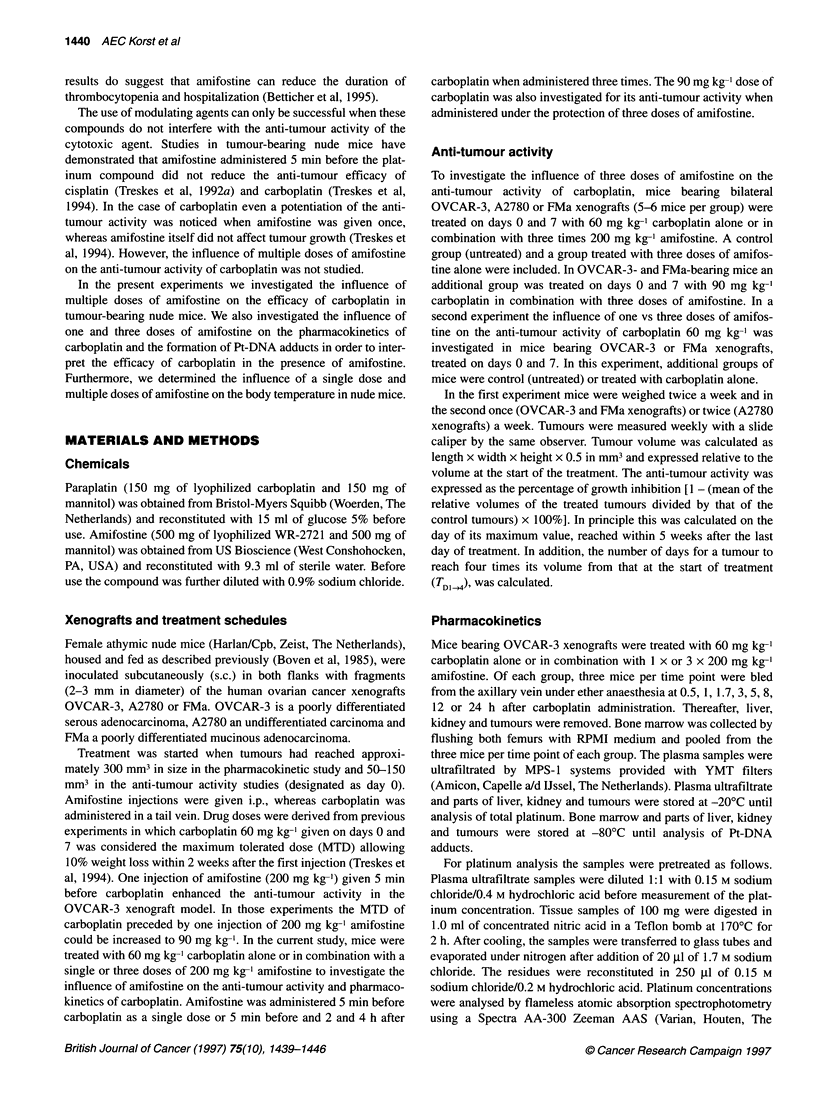

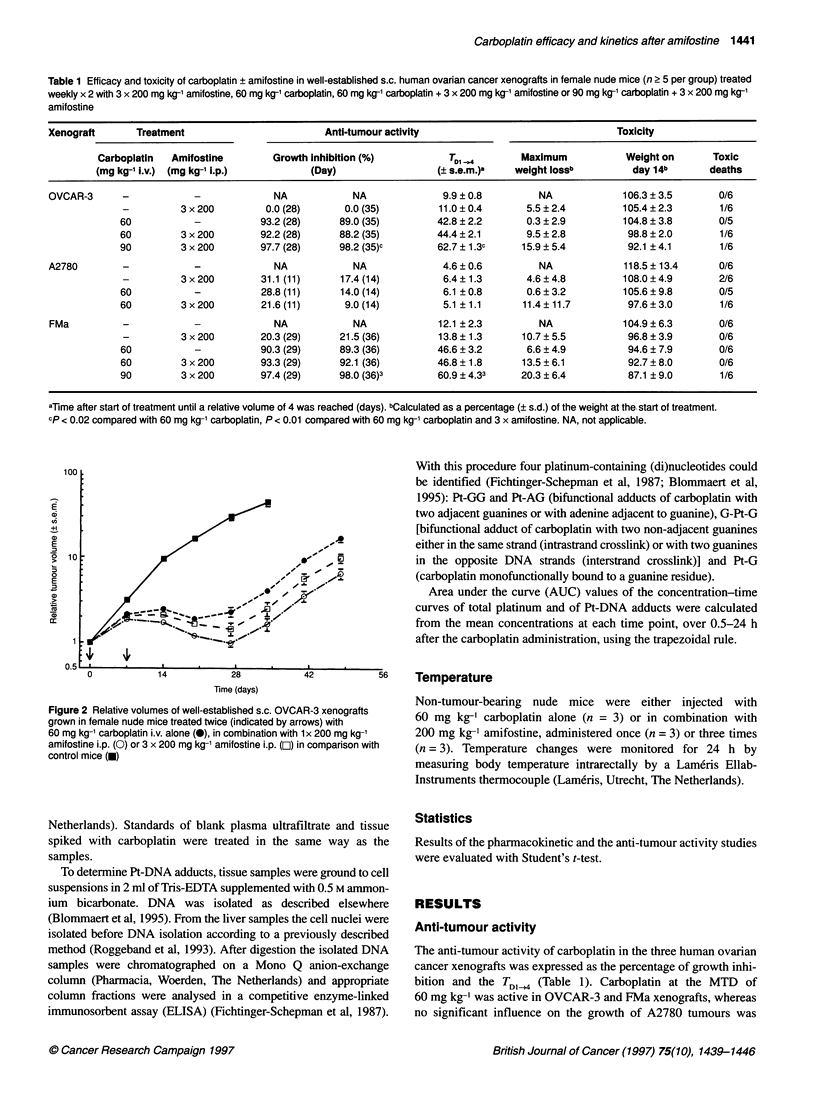

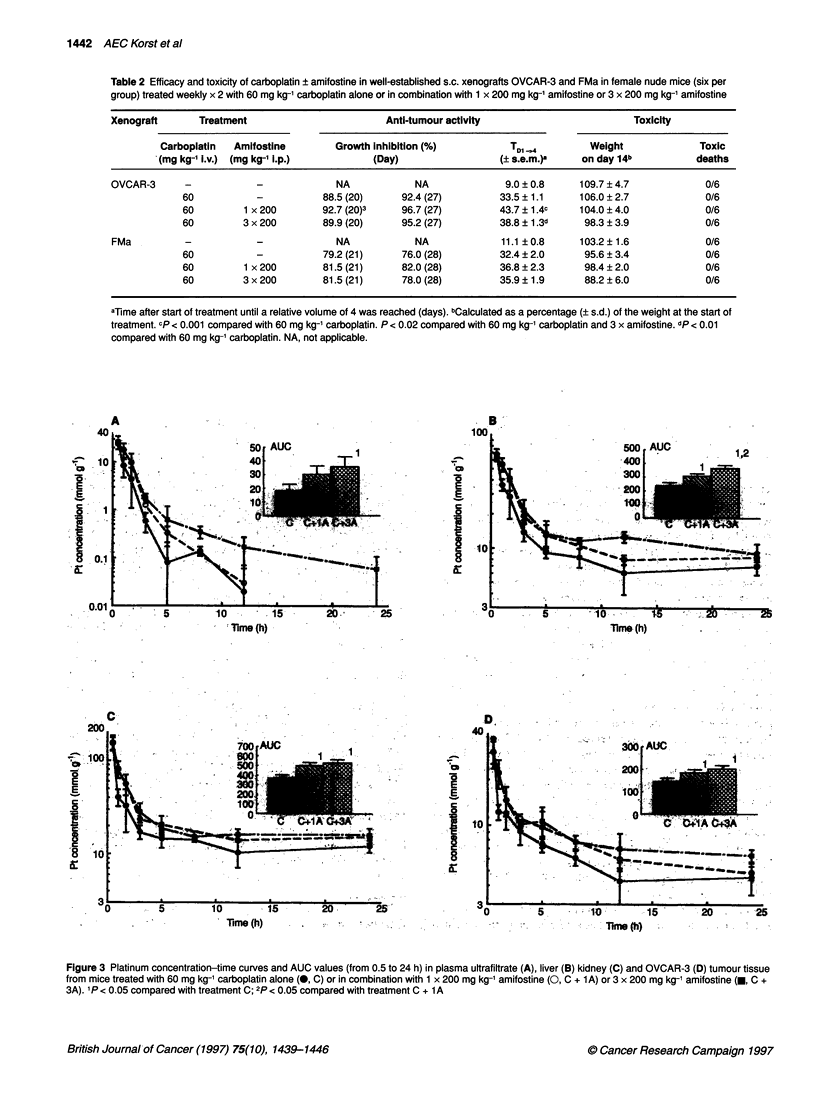

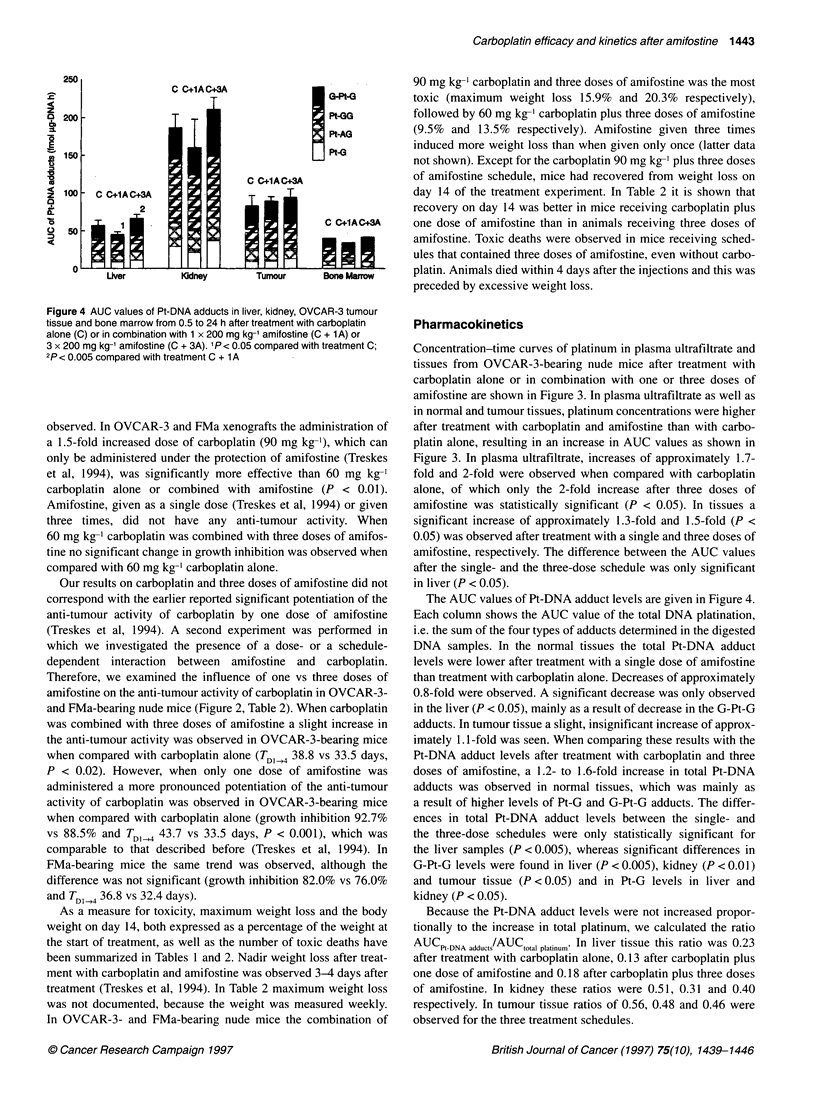

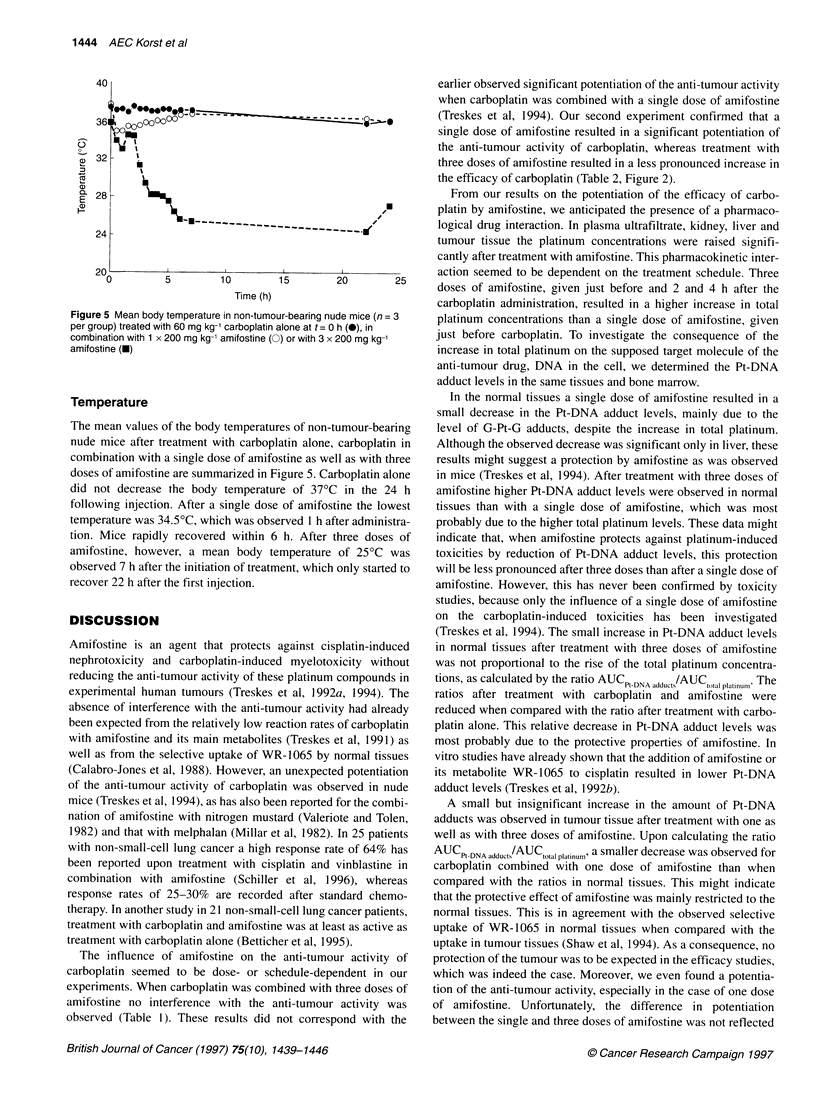

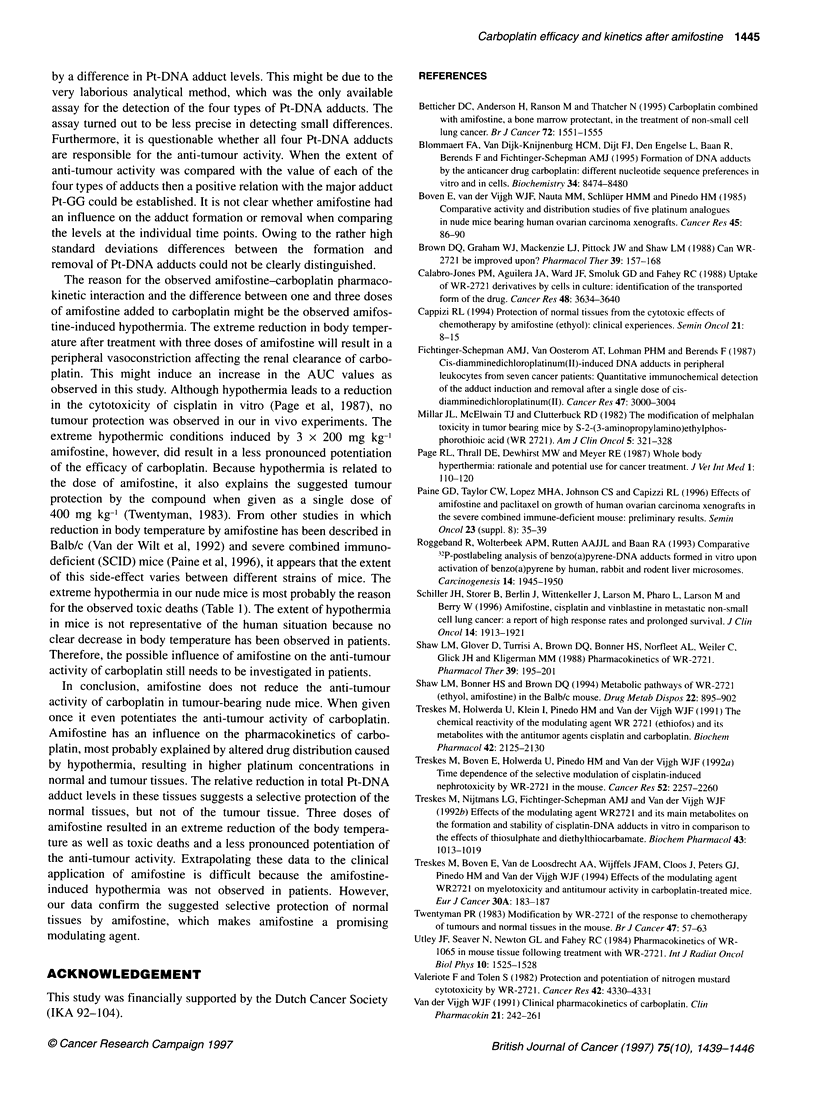

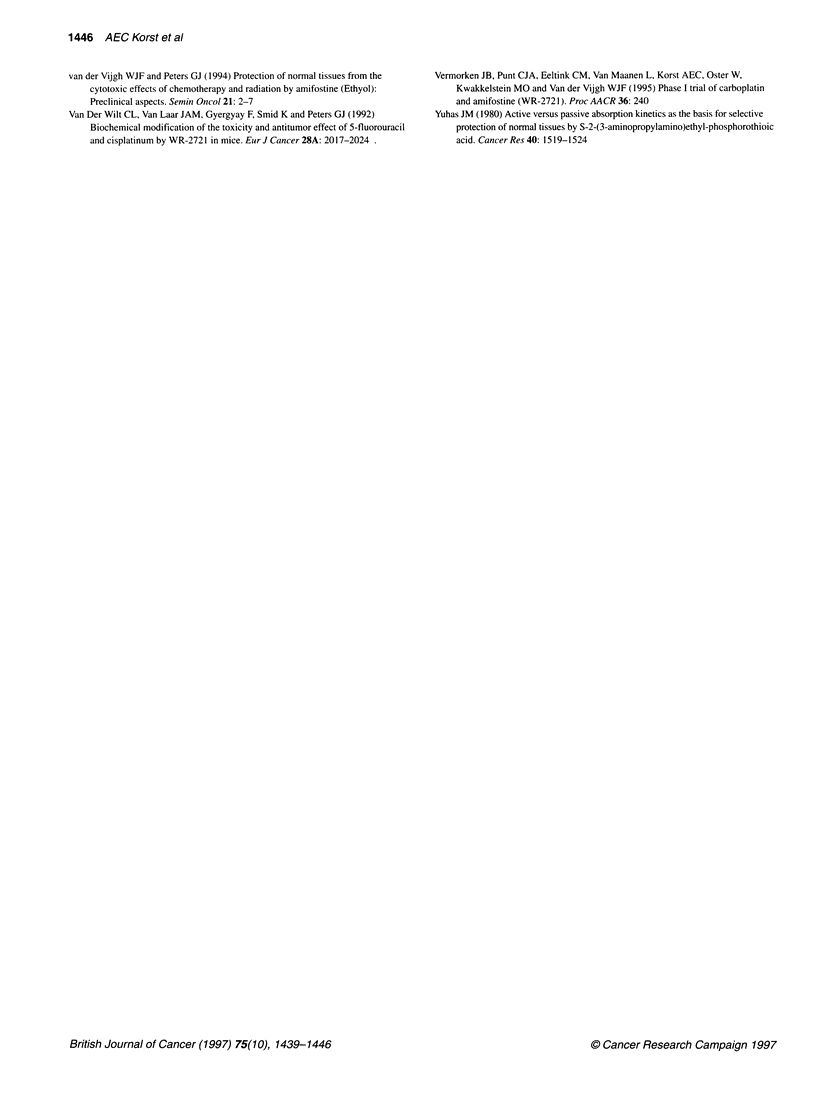


## References

[OCR_00787] Betticher D. C., Anderson H., Ranson M., Meely K., Oster W., Thatcher N. (1995). Carboplatin combined with amifostine, a bone marrow protectant, in the treatment of non-small-cell lung cancer: a randomised phase II study.. Br J Cancer.

[OCR_00792] Blommaert F. A., van Dijk-Knijnenburg H. C., Dijt F. J., den Engelse L., Baan R. A., Berends F., Fichtinger-Schepman A. M. (1995). Formation of DNA adducts by the anticancer drug carboplatin: different nucleotide sequence preferences in vitro and in cells.. Biochemistry.

[OCR_00799] Boven E., van der Vijgh W. J., Nauta M. M., Schlüper H. M., Pinedo H. M. (1985). Comparative activity and distribution studies of five platinum analogues in nude mice bearing human ovarian carcinoma xenografts.. Cancer Res.

[OCR_00806] Brown D. Q., Graham W. J., MacKenzie L. J., Pittock J. W., Shaw L. M. (1988). Can WR-2721 be improved upon?. Pharmacol Ther.

[OCR_00810] Calabro-Jones P. M., Aguilera J. A., Ward J. F., Smoluk G. D., Fahey R. C. (1988). Uptake of WR-2721 derivatives by cells in culture: identification of the transported form of the drug.. Cancer Res.

[OCR_00820] Fichtinger-Schepman A. M., van Oosterom A. T., Lohman P. H., Berends F. (1987). cis-Diamminedichloroplatinum(II)-induced DNA adducts in peripheral leukocytes from seven cancer patients: quantitative immunochemical detection of the adduct induction and removal after a single dose of cis-diamminedichloroplatinum(II).. Cancer Res.

[OCR_00828] Millar J. L., McElwain T. J., Clutterbuck R. D., Wist E. A. (1982). The modification of melphalan toxicity in tumor bearing mice by s-2-(3-aminopropylamino)- ethylphosphorothioic acid (WR 2721).. Am J Clin Oncol.

[OCR_00833] Page R. L., Thrall D. E., Dewhirst M. W., Meyer R. E. (1987). Whole-body hyperthermia. Rationale and potential use for cancer treatment.. J Vet Intern Med.

[OCR_00838] Paine G. D., Taylor C. W., Lopez M. H., Johnson C. S., Capizzi R. L. (1996). Effects of amifostine and paclitaxel on growth of human ovarian carcinoma xenografts in the severe combined immune-deficient mouse: preliminary results.. Semin Oncol.

[OCR_00844] Roggeband R., Wolterbeek A. P., Rutten A. A., Baan R. A. (1993). Comparative 32P-postlabeling analysis of benzo[a]pyrene--DNA adducts formed in vitro upon activation of benzo[a]pyrene by human, rabbit and rodent liver microsomes.. Carcinogenesis.

[OCR_00850] Schiller J. H., Storer B., Berlin J., Wittenkeller J., Larson M., Pharo L., Larson M., Berry W. (1996). Amifostine, cisplatin, and vinblastine in metastatic non-small-cell lung cancer: a report of high response rates and prolonged survival.. J Clin Oncol.

[OCR_00862] Shaw L. M., Bonner H. S., Brown D. Q. (1994). Metabolic pathways of WR-2721 (ethyol, amifostine) in the BALB/c mouse.. Drug Metab Dispos.

[OCR_00857] Shaw L. M., Glover D., Turrisi A., Brown D. Q., Bonner H. S., Norfleet A. L., Weiler C., Glick J. H., Kligerman M. M. (1988). Pharmacokinetics of WR-2721.. Pharmacol Ther.

[OCR_00885] Treskes M., Boven E., van de Loosdrecht A. A., Wijffels J. F., Cloos J., Peters G. J., Pinedo H. M., van der Vijgh W. J. (1994). Effects of the modulating agent WR2721 on myelotoxicity and antitumour activity in carboplatin-treated mice.. Eur J Cancer.

[OCR_00865] Treskes M., Holwerda U., Klein I., Pinedo H. M., van der Vijgh W. J. (1991). The chemical reactivity of the modulating agent WR2721 (ethiofos) and its main metabolites with the antitumor agents cisplatin and carboplatin.. Biochem Pharmacol.

[OCR_00892] Twentyman P. R. (1983). Modification by WR 2721 of the response to chemotherapy of tumours and normal tissues in the mouse.. Br J Cancer.

[OCR_00896] Utley J. F., Seaver N., Newton G. L., Fahey R. C. (1984). Pharmacokinetics of WR-1065 in mouse tissue following treatment with WR-2721.. Int J Radiat Oncol Biol Phys.

[OCR_00901] Valeriote F., Tolen S. (1982). Protection and potentiation of nitrogen mustard cytotoxicity by WR-2721.. Cancer Res.

[OCR_00928] Yuhas J. M. (1980). Active versus passive absorption kinetics as the basis for selective protection of normal tissues by S-2-(3-aminopropylamino)-ethylphosphorothioic acid.. Cancer Res.

[OCR_00905] van der Vijgh W. J. (1991). Clinical pharmacokinetics of carboplatin.. Clin Pharmacokinet.

[OCR_00913] van der Vijgh W. J., Peters G. J. (1994). Protection of normal tissues from the cytotoxic effects of chemotherapy and radiation by amifostine (Ethyol): preclinical aspects.. Semin Oncol.

[OCR_00918] van der Wilt C. L., van Laar J. A., Gyergyay F., Smid K., Peters G. J. (1992). Biochemical modification of the toxicity and the anti-tumour effect of 5-fluorouracil and cis-platinum by WR-2721 in mice.. Eur J Cancer.

